# Deep Features for Training Support Vector Machines

**DOI:** 10.3390/jimaging7090177

**Published:** 2021-09-05

**Authors:** Loris Nanni, Stefano Ghidoni, Sheryl Brahnam

**Affiliations:** 1 Department of Information Engineering (DEI), University of Padova, 35131 Padova, Italy; stefano.ghidoni@unipd.it; 2 Information Technology and Cybersecurity (ITC), Missouri State University, 901 S National, Springfield, MO 65804, USA; sbrahnam@missouristate.edu

**Keywords:** deep learning, transfer learning, global mean thresholding pooling, support vector machines, ensemble of descriptors

## Abstract

Features play a crucial role in computer vision. Initially designed to detect salient elements by means of handcrafted algorithms, features now are often learned using different layers in convolutional neural networks (CNNs). This paper develops a generic computer vision system based on features extracted from trained CNNs. Multiple learned features are combined into a single structure to work on different image classification tasks. The proposed system was derived by testing several approaches for extracting features from the *inner layers* of CNNs and using them as inputs to support vector machines that are then combined by sum rule. Several dimensionality reduction techniques were tested for reducing the high dimensionality of the inner layers so that they can work with SVMs. The empirically derived generic vision system based on applying a discrete cosine transform (DCT) separately to each channel is shown to significantly boost the performance of standard CNNs across a large and diverse collection of image data sets. In addition, an ensemble of different topologies taking the same DCT approach and combined with global mean thresholding pooling obtained state-of-the-art results on a benchmark image virus data set.

## 1. Introduction

Extracting salient descriptors from images is the mainstay of many computer vision systems. Typically, these handcrafted descriptors are tailored to overcome specific problems in image classification with the goal being to achieve the best classification accuracy possible while maintaining computational efficiency. Some descriptors such as the scale invariant feature transform (SIFT) [1] are valued for their robustness, but they can be too computationally expensive for practical purposes. As a consequence, variants of popular handcrafted descriptors such as some fast variants of SIFT [2] continue to be created in an attempt to overcome inherent shortcomings.

In contrast to computer vision systems that rely on the extraction of handcrafted descriptors are those that depend on deep learners [3] as exemplified in computer vision by convolutional neural networks (CNNs). Deep learning involves designing complex networks composed of specialized layers, and the descriptors or features calculated by these layers are learned from the training samples [4]. Layers in deep learners such as CNNs are known to discover many low-level representations of data in early stages that become useful to subsequent layers that are in charge of providing higher-level features representing the semantics of the data [5]. Close to the input, edges and texture are usually detected [6]. Higher up, features like contours and image patches are discerned. Layer by layer, representations of the data in deep learners become more and more complex. An advantageous characteristic of these deep features is that they are generalizable. Once extracted, they can be treated like other handcrafted features in traditional computer vision systems and applied to many different image problems.

Interest in research investigating feature sets extracted from different layers of pre-trained CNNs has grown in recent years. Lower level features extracted from sets of CNN topologies have been explored in [7,8] as well as top layers in [9,10,11]. In [7], for example, features were extracted from the lower layers of pretrained CNNs and trained on support vector machines (SVMs) [12]. In [9], images are represented as strings of CNN features taken from the top layer with similarities when compared with novel distance measures. In [13], convolutional features were extracted and used as a filter bank. In [14], deep activation features were extracted from local patches at multiple scales with convolutional features taken from the seventh layer of a CNN trained on ImageNet. In [10], features were extracted from the sixth and seventh fully connected (FC) layers of two different topologies. In [15,16], features were extracted from the last convolutional layers of a CNN and in [16], combined with the fully connected (FC) layer. In [17], images are represented using five convolutional layers and two FC layers. Similarly, in [18], convolutional features were extracted from multiple layers combined with FC features. In [19], features were extracted from the penultimate layers of pretrained CNNs and merged with the outputs of the deep layers and CNN scores. Finally, in [20], features were investigated layer by layer and were discovered to provide quality information about the texture of images at multiple depths.

The literature is replete with studies that have investigated training SVMs with features extracted from CNNs (see, for example, [7,10,19,21,22,23,24,25]). SVMs are a preferred classifier mainly because of the computational resources needed to fine-tune CNNs. Replacing the SoftMax or the FC layer of a CNN with SVM greatly reduces training time and has been proven to produce excellent results. Most studies investigating SVMs in this way have pulled features from the last layers of CNNs, with fewer, such as [7], examining lower-level features. studies focused on training SVMs with features extracted from the inner layers have been neglected. Extracting features from the inner layers of CNNs poses a difficulty because such features are characterized by high dimensionality, making them unsuitable for training statistical classifiers like SVM.

This work aims to exploit both the deeper and shallower layers of pretrained CNNs for representing images with fixed-length feature vectors that can then be trained on a set of SVMs. To reduce the dimensionality of features taken from the inner layers, experiments are run that test the following approaches (see Section 2.2, Section 2.3, Section 2.4, Section 2.5 and Section 2.6 for details):

- Classic dimensionality reduction methods: viz., discrete cosine transform (DCT) and principal component analysis (PCA);
- Feature selection approaches (chi-square feature selection);
- Extraction of descriptors from local binary patterns (LBP), followed by feature selection;
- Co-occurrence among elements of the channels of inner layers;
- Global pooling measurements: global entropy pooling (GEP) and global mean thresholding pooling (GMTP);
- Sequential forward floating selection (SFFS) of layers and classifiers.

Experiments demonstrate that combining feature sets extracted from inner and outer CNN layers and applying as many different dimensionality reduction techniques as needed obtains close to, if not state-of-the-art, results on an extensive collection of cross-domain image data sets. The best ensemble tested in this work is based on DCT that is applied separately on each channel; this new method is shown in the experimental section to outperform standard CNNs as well as a global DCT approach across the collection of image data sets. Moreover, the ensemble (DCT+GMTP)-2, which combines our approach with GMTP, obtained state-of-the-art results on a virus benchmark data set, outperforming standard CNNs (DenseNet201 and ResNet50) and methods based on training SVMs on features extracted from the typical top layers of these networks. Performance differences were verified using the Wilcoxon signed-rank test, and all experiments can be replicated using the MATLAB source code available at https://github.com/LorisNanni (accessed on 9 February 2020).

## 2. Methods

### 2.1. Feature Extraction from CNNs

**Figure 1:**
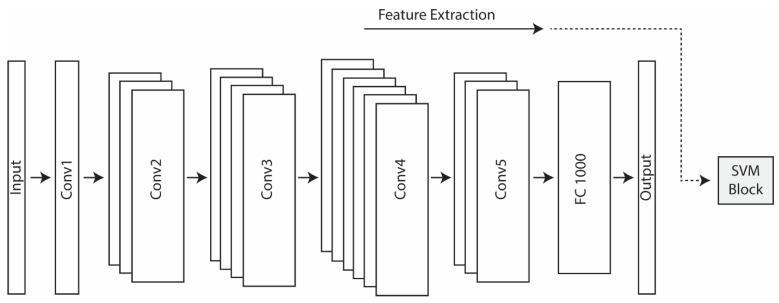
The five convolutional layers (Conv1-5) and the last fully connected layer (FC) of ResNet50, pretrained on ImageNet. Features extracted from the inner layers of ResNet50 were fed into the SVM block.

**Figure 2:**
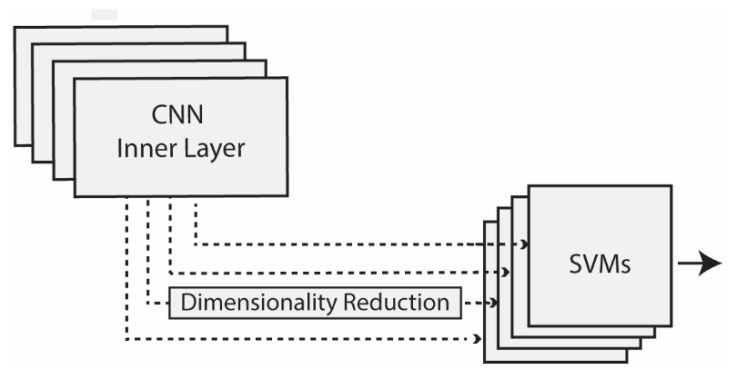
Feature extraction from inner layers. The output of each layer was treated as a feature vector with dimensionality reduction methods applied depending on the vector size (>5000). All vectors were then processed by a separate SVM and summed for a final decision.

In this work, we extracted features from CNNs [26] that were pretrained on the ImageNet dataset [27]. These features were taken from multiple layers of a CNN and then individually trained on separate SVMs (see Figure 1 and Figure 2). The CNN architectures investigated in this study were GoogleNet (Inception IV) [28], ResNet50 [29], and Densenet201 [30]. GoogleNet, winner of the ImageNet Large-Scale Visual Recognition Challenge (ILSVRC) in 2014, is a CNN with 22 layers. To create deeper layers, GoogleNet uses 1×1 convolution and global average pooling. ResNet50, the winner of the ILSVRC2015 contest, is a CNN with 50 layers. To overcome the vanishing gradient problem in deep networks, ResNet incorporates a residual connection. DenseNet201 is extremely deep with 201 layers. This architecture replaces the residual connection with densely connected convolutional layers that are concatenated rather than added to each other as with ResNet. All layers are interconnected in DenseNet, a technique that produces strong gradient flow and that shares low-level information across the entire network.

Unlike many other studies focused on the extraction of features from the output layer, we examined features extracted from deeper layers, as in [19]. The layers considered for extracting features were selected starting from the middle layer of the network and then by considering 1 layer after every 10, going toward the output layer with the last four layers always considered. As deep layers encode high-dimension features, dimensionality reduction methods were also used, as shown in Figure 2, depending on the feature size (i.e., when more than 5000 features were extracted by a given layer).

All configurations were investigated considering the possible combinations of the following elements:

- Tuning (with/without): Either the CNN used to extract features was pretrained on ImageNet without any tuning, or it was tuned on the given training set;
- Scope of dimensionality reduction (local/global): Either dimensionality reduction was performed separately on each channel of a layer (with results combined), or reduction was applied to the whole layer;
- PCA postprocessing (with/without): Either PCA projection was performed after dimensionality reduction, or PCA was not applied.

The dimensionality reduction methods considered in this work are presented in the remainder of this section.

### 2.2. Feature Reduction Transforms (PCA and DCT)

Dimensionality is reduced by applying two classic transforms: PCA and DCT.

PCA [31] is a well-known, unsupervised technique that projects high-dimensional data into a lower-dimensional subspace. This is accomplished by mapping the original feature vectors into a smaller number of uncorrelated directions that preserve the global Euclidean structure.

The DCT transform [32] balances information packing and computational complexity. DCT components tend to be small in magnitude because the most important information lies in the coefficients with low frequencies. As with PCA, removing small coefficients produces small errors when the transform is reversed to reconstruct the original images.

### 2.3. Chi-Square Feature Selection (CHI)

Univariate feature ranking for classification using chi-square tests is a popular feature selection method. In the experimental section, CHI is the label used for chi-square feature selection.

The chi-square test in statistics tests the independence between two events A and B. If P(AB) = P(A)P(B), then the two events are said to be independent. The same holds when P(A|B) = P(A) and P(B|A) = P(B).

The formula for the chi-square test is

(1) $$X_{c}^{2}=\sum {\left(O_{i}-E_{i}\right)}^{2}/E_{i},$$

where c are the degrees of freedom, O are the observed values, and $E_{i}$ are the expected values. The degrees of freedom are the maximum number of logically independent values (the total number of observations minus the number of imposed constraints).

Applied to feature selection, chi-square is calculated between every feature variable and the target variable (the occurrence of the feature and the occurrence of the class). If the feature variable is independent of the class, it is discarded; otherwise, it is selected.

### 2.4. Local Binary Patterns

This approach to feature reduction is based on a uniform local binary pattern (LBP), a popular texture descriptor. LBP is defined across each pixel value ($I_{c}$) on a local circular neighborhood of radius R of size N pixels, thus

(2) $$LBP\left(N,R\right)=\sum_{n=0}^{N-1}s\left(I_{n}-I_{c}\right)2^{N},$$

where s(x)=1 if x≥0 and equal to 0 otherwise. A histogram of the resulting binary numbers describes the texture of a given image.

When calculating (2), two types of patterns are distinguished, those with less than three transitions between 0 and 1, known as *uniform* patterns, and the remainder, which are called *nonuniform*.

In this work, N=8, R=1, and only uniform patterns, as already mentioned, are considered. After LBP extraction from each channel of a CNN layer, dimensionality was reduced with the chi-square feature selection method. In the experimental section, the dimensionality method based on LBP combined with chi-square feature selection is labeled LBPCHI.

### 2.5. Deep Co-Occurrence of Deep Representation (CoOC)

A CoOC can be obtained from a deep convolutional layer, as proposed in [16]. A co-occurrence is said to occur when the values of two separate activations located inside a given region are greater than a certain threshold. The resulting representation is a tensor with the same dimensions as the activation tensor and can be implemented with convolutional filters.

A convolutional filter can be defined as $F\in {\mathbb{R}}^{D\times D\times S\times S}$ where D is the number of channels in the activation tensor and where the size of the co-occurrence window is S=2·𝓇+1 with 𝓇, the radius, defining the co-occurrence region. Filters are initially set to 1, except for the filter that is related to a given channel; such a filter is initialized to 0 or some very small value of ε.

Given the activation tensor A of size M×N with D channels where $A\in {\mathbb{R}}^{M\times N\times D}$ and where A is the last convolution operator in a CNN, co-occurrence tensor $C_{T}\in {\mathbb{R}}^{M\times N\times D}$ can be considered a convolution between the activation tensor after thresholding the co-occurrence filter, thus

(3) $$C_{T}=\left(A_{\rho_{A}}*F\right)\cdot \rho_{A}$$

where $A_{\rho_{A}}=A\cdot \rho_{A},$ with $\rho_{A}\in {\mathbb{R}}^{M\times N\times D}$ and $\rho_{A}=A>\bar{A}$ with $\bar{A}$ the average mean of the activation map produced after the last convolutional layer. In other words, given the activation $\alpha_{i,j}^{k}$

(4) $$\rho_{A}\left(i,j,k\right)=\{\begin{array}{c} 1,\;if\;\alpha_{i,j}^{k}>\frac{1}{M\cdot N\cdot D}\sum_{i=1}^{M}\sum_{j=1}^{N}\sum_{k=1}^{D}\alpha_{i,j}^{k}.\\ 0,\;\mathrm{otherwise}. \end{array}$$

For pseudo-code, see [16].

### 2.6. Global Pooling Measurements

The input to a global pooling layer is a set of $n_{A^{\left(l\right)}}$ activation maps computed previously by layer l, and the output is one global measurement $g(A_{i}^{\left(l\right)}$) for each activation map $A_{i}^{\left(l\right)}(1\leq i\leq n_{A^{\left(l\right)}}$). The $A^{\left(l\right)}$ measurements then become the inputs to an FC layer. In [20], these pooling measurements were transformed into feature vectors. Two global pooling measurements are used for feature extraction in the experiments presented here: global entropy pooling (GEP) and global mean thresholding pooling (GMTP).

GEP computes the entropy value of $A_{i}^{\left(l\right)}$. Given the probability distribution $p_{i}^{\left(l\right)}$ of $A_{i}^{\left(l\right)}$, calculated first by normalizing values to [0, 255] and then by computing a histogram from the normalized activation map using 255 bins, $p_{i}^{\left(l\right)}$ is simply the resulting histogram divided by the sum of its elements:

(5) $$\sum_{j}p_{i}^{\left(l\right)}\left[j\right]=1,\;\left(0\leq j\leq 255\right)$$

Thus, GEP is defined as:

(6) $$\mathrm{GEP}\left(A_{i}^{\left(l\right)}\right)=-\sum_{j}p_{i}^{\left(l\right)}\left[j\right]\ln\left(p_{i}^{\left(l\right)}\left[j\right]\right)$$

Unlike GEP, GMPT includes more layer information in the feature extraction process. To compute GEP, a threshold $T_{g}^{\left(l\right)}$ must be obtained by averaging the value of the entire set of activation maps $A^{\left(l\right)}:$

(7) $$T_{g}^{\left(l\right)}=\frac{\sum_{i}\sum_{v}\sum_{u}(A_{i}^{\left(l\right)}\left[v,u\right])}{n_{A^{\left(l\right)}}*h_{A^{\left(l\right)}}*w_{A^{\left(l\right)}}}$$

where v and u are an element’s position in the i-th activation map computed previously by layer l. Whereas $n_{A^{\left(l\right)}}$, as already noted, represents the number of activation maps, $h_{A^{\left(l\right)}}$ and $w_{A^{\left(l\right)}}$ are the height and width of each map. Thus, GMPT is the proportion of elements in each $A^{\left(l\right)}$ with values below threshold $T_{g}^{\left(l\right)}$.

### 2.7. Sequential Forward Floating Selection of Layers/Classifiers

In some of the experiments presented in this work, we examine the performance of a layer selection method (i.e., a classifier selection procedure) using sequential forward floating selection, as described in [33]. Selecting classifiers using SFFS is performed by including models in the final ensemble that produce the highest increments of performance compared to an existing subset of models. A backtracking step replaces the worst model from the actual ensemble, using the better-performing model. As SFFS requires a training phase to select the best models for the task, we performed a leave-one-out data set selection protocol.

The CNNs were trained with a batch size of 30 and a learning rate (LR) of 0.0003 for 20 epochs (the last FC layer had a LR 20 times larger). Images were augmented with random reflections on both axes and two independent random rescales of both axes by two factors uniformly sampled in [1,2] (using standard MATLAB data augmentation procedures).

## 3. Experimental Results

This section describes the experimental results on 12 publicly available medical image data sets. Section 3.1 provides a short discussion of the data sets. Data set names and abbreviations have been shortened to two letters to minimize clutter and increase clarity within the tables reporting classification results. In Section 3.2, experimental results are presented. The reader may wish to consult Appendix A for the meanings of the acronyms used in this paper and in the construction of the tested ensembles.

### 3.1. Discription of Data Sets

Our proposed system was tested on the following data sets:

- CH (CHO data set [34]), containing 327 fluorescence microscope 512 × 382 images of Chinese hamster ovary cells divided into five classes;
- HE (2D HeLa data set [34]), containing 862 fluorescence microscopy 512 × 382 images of HeLa cells stained with various organelle-specific fluorescent dyes. The images were divided into 10 classes of organelles;
- RN (RNAi data set [35]), containing 200 fluorescence microscopy 1024 × 1024 TIFF images of fly cells (*D. melanogaster*) divided into 10 classes;
- MA (C. Elegans Muscle Age data set [35]), containing 237 1600 × 1200 images for classifying the age of the nematode given 25 images of *C. elegans* muscles collected at four ages;
- TB (Terminal Bulb Aging data set [35]), the companion data set to MA and contains 970 768 × 512 images of *C. elegans* terminal bulbs collected at seven ages;
- LY (Lymphoma data set [35]), containing 375 1388 × 1040 images of malignant lymphoma representative of three types;
- LG (Liver Gender Caloric Restriction (CR) data set [35]), containing 265 1388 × 1040 images of liver tissue sections from six-month old male and female mice on a CR diet;
- LA (Liver Aging Ad-libitum data set [35]), containing 529 1388 × 1040 images of liver tissue sections from female mice on an ad-libitum diet divided into four classes representing the age of the mice;
- BGR (Breast Grading Carcinoma [36]): This is a Zenodo data set (record: 834910#.Wp1bQ-jOWUl) that contains 300 1280 × 960 annotated histological images of 21 patients with invasive ductal carcinoma of the breast representing three classes/grades;
- LAR (Laryngeal data set [37]): This is a Zenodo data set (record: 1003200#.WdeQcnBx0nQ) containing 1320 1280 × 960 images of 33 healthy and early-stage cancerous laryngeal tissues representative of four tissue classes;
- LO (Locate Endogenous data set [38]), containing 502 768 × 512 images of endogenous cells divided into 10 classes. This data set is archived at https://integbio.jp/dbcatalog/en/record/nbdc00296 (accessed on 9 January 2021).
- TR (Locate Transfected data set [38]) is a companion data set to LO and contains 553 768 × 512 images divided into the same 10 classes as LO but with the addition of 1 more class for a total of 11 classes.

Data sets 1–8 can be found at https://ome.grc.nia.nih.gov/iicbu2008/ (accessed on 9 January 2021), and data sets 9–10 are on Zenodo and can be accessed by the record number provided in parentheses in the data set descriptions. Data sets 10 and 12 are available upon request.

The five-fold cross-validation protocol was applied to all data sets except for LAR, which uses a three-fold protocol. Although the size of the original images is provided above in the data set descriptions, all images were resized to fit the input size for the given CNN model.

### 3.2. Experimental Results

In our experiments, we obtained better results tuning the CNN on each training set without PCA processing and with application of the methods locally (i.e., separately on each channel of a given layer). For this reason, most of the results reported in the following tables for the dimensionality reduction methods (unless otherwise specified) are based on tuning the CNNs without PCA postprocessing and with the local application of methods. As noted in the introduction, a well-known statistical measure for comparing data, the Wilcoxon signed-rank test [39], also known as the Wilcoxon signed-rank sum test, was the measure used to validate experiments.

Reported in Table 1 is the performance of all the approaches using ResNet50, and reported in Table 2 are the most interesting results using GoogleNet. The row labeled *CNN* in Table 1 and Table 2 reports the performance obtained using a standard standalone CNN, either ResNet50 (Table 1) or GoogleNet (Table 2). The label *TunLayer-x* represents features extracted for SVM training using the x-to-last layer of the network tuned on the given training set. The best performance for TunLayer-x was obtained with x=3; we also report, for comparison purposes, the performance of Layer-3 on the CNN pretrained on ImageNet without tuning on the given data sets. The label *TunFusLayer* is the fusion by sum rule of the TunLayer-x classifiers. Row X+Y indicates the sum rule between approaches X and Y, and the method named *g-DC* is DC applied globally, as in [19].

The SVM classifiers were tested using LibSVM and fitecoc. LibSVM (available at https://www.csie.ntu.edu.tw/~cjlin/libsvm/ accessed on 9 January 2021) is an integrated SVM library available for many languages, including MATLAB, that supports one-class and multiclass SVM classification; fitecoc is a MATLAB module that supports multiclass versions of SVMs as well as other classifiers. With the LibSVM library, the SVM hyperparameters were not optimized; rather, we used the generic settings: radial basis function kernel with C = 1000 and gamma = 0.1. Generic settings were also used for fitecoc, which performed better, except for CoOC, and when the CNN was not tuned. For the last four layers, we trained SVM, using the original features.

Feature vectors produced by a given layer of the CNN with a dimensionality higher than 5000 were processed by applying the dimensionality reduction techniques in the following ways:

- DoOC, GEP, and GMTP used a single value extracted from each channel (see details in the previous section);
- For the other approaches, the method was first applied separately on each channel; next, 1000/(number of channels) features were extracted from each channel;
- For g-DCT, all features from all channels were first concatenated; next, they were reduced to a 1000-dimension vector by applying DCT;

In the following tables, some classifiers are labeled as:

- *Ens15CNN*, an ensemble, which is the sum rule among 15 standard ResNet50 CNNs or 15 standard GoogleNets. This is a baseline approach since our method is an ensemble of classifiers;
- (DCT+GMTP)-2, the combined approach of DCT plus GMTP, where the two last layers of the CNN are not used for feeding SVM. Notice that DCT and GMTP were extracted considering two different trainings of the CNN (this was done in order to increase the diversity of the features extracted by the two methods);
- SFFS(X) means that we combined by sum rule X SVMs, selected using the method detailed in Section 2.7.

For GoogleNet, only the most interesting approaches that were reported for ResNet50 were tested; this was done to reduce computation time.

An analysis of the results reported in Table 1 and Table 2 leads to the following set of observations:

- DCT clearly outperformed g-DCT on both GoogleNet and ResNet50 with a *p*-value of 0.0001 (the lower the p-value, the greater the statistical significance). Applying DCT separately on each channel boosted performance with respect to a single application of DCT on the whole layer;
- The best methods for reducing the dimensionality of the inner layers were DC, PCA, GMTP, and GEP;
- On average, the best approach was given by (DCT + GMTP)-2, i.e., by the sum rule between DCT and GMTP;
- On average, discarding the SVMs trained with the two last layers slightly improved performance;
- DCT outperformed (*p*-value 0.01) on any TunLayer-x; this implies that the inner layers are also useful on the tuned networks.
- Using ResNet50 as the classifier produced an average accuracy of 85.5%; this performance was boosted to 88.6% using the proposed gDCT approach. The new, channel-based DCT method further improved performance to 93.1%; finally, the best performance was obtained by ensemble (DCT + GMTP)-2, which reached an accuracy of 93.9%. A similar progression of performance enhancement was obtained using GoogleNet as the CNN classifier.

Both TunLayer-3 and DCT strongly outperformed (*p*-value 0.01) CNN on all the tested data sets. Using GoogleNet/ResNet50 directly to classify images did not maximize performance, probably due to overfitting given the size of the training sets. We also trained an SVM classifier on each of the 10 layers. Considering the size of GoogleNet and ResNet50, using larger CNNs with so many layers would not have been the best choice.

**Figure 3:**
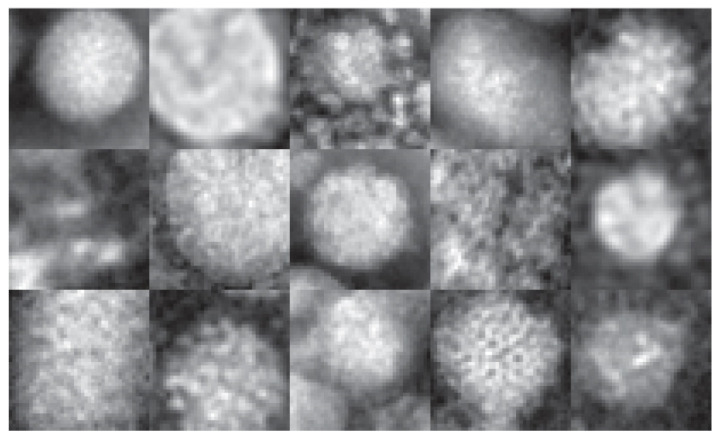
Examples of all 15 classes in the virus benchmark [40] (moving left to right, with class 1 top left and class 15 bottom right).

To test the generalizability of our approach, Table 3 reports experiments run on a popular virus benchmark [40] (see Figure 3 for examples). This data set contains 1500 41 × 41 transmission electron microscopy (TEM) images of viruses belonging to 15 species of viruses and is divided into two different data sets: (1) the *object scale* data set, so named because the radius of every virus in each image is 20 pixels, and (2) the *fixed scale* data set, so called because each virus image is represented such that the size of 1 pixel corresponds to 1 nm. The first data set used in the following experiments is publicly available. The second is proprietary, so it is unavailable for testing due to copyright issues. It is the *object scale* data set that is widely reported in the literature.

Regarding object scale, two networks were trained: DenseNet201, which is the network providing the best performance in the literature on the object scale dataset, and ResNet50 (because a large number of relevant papers report results using this network). Both CNNs were trained for 50 epochs with all other parameters the same as those noted in the tests reported above.

In Table 3, we report the performance obtained using both LibSVM and fitecoc classifiers. DenseNet produced better performance using LibSVM. To reduce computation time, the combination (DCT+PCA+GMTP)-2 was not run on ResNet because when coupled with DenseNet, it obtained performance similar to (DCT+GMTP)-2. The last column of Table 3 reports the fusion by sum rule between the two CNNs before the sum of the scores of each ensemble was normalized by dividing the score by the number of trained SVMs.

Finally, in Table 4, we compare our approach with the best performance reported in the literature. As can be observed in Table 4, our proposed method obtained state-of-the-art performance. In [40], the reported performance was obtained using the *fixed scale* data set. As that data set is not publicly available, comparisons with [40] cannot be made. By combining features computed on the *object scale* data set with the *fixed scale* data sets, an accuracy of 87.0% was obtained in their work.

## 4. Conclusions

The objective of this work was to explore the power of using both the intermediate and the last layers of three pretrained CNNs to evaluate features with fixed-length feature vectors trained on an ensemble of SVMs. To overcome the high dimensionality of the features extracted from the inner layers, our experiments investigated many different dimensionality reduction techniques including two classic feature transforms (DCT and PCA), a feature selection approach (chi-square feature selection), a representation based on the co-occurrence among elements of the channels of inner layers, and a texture descriptor (LBP), followed by feature selection.

The best ensemble reported here was shown to significantly boost the performance of standard CNNs on a large and diverse group of image data sets as well as on a popular benchmark virus data set, in which the best ensemble obtained state-of-the-art performance.

In future works, we plan on combining this approach with other deep neural networks and testing different methods for representing the inner layers in a compact way for training on SVMs.

## Figures and Tables

**Table 1:** Performance using ReNet50 (Bold face indicates best performances).

Method	ResNet												Avg
	CH	HE	LO	TR	RN	TB	LY	MA	LG	LA	BG	LAR	
CNN	93.2	91.0	91.6	94.2	56.5	62.5	76.3	90.8	92.6	88.9	87.3	91.6	85.5
Ens15CNN	99.0	96.0	98.4	98.5	66.0	70.2	92.2	95.8	99.3	98.6	94.0	94.7	91.9
TunLayer-3	98.1	93.8	97.4	96.3	74.0	68.2	86.9	94.1	100	98.2	91.3	91.4	91.2
TunLayer-2	96.0	91.2	95.4	95.0	70.5	65.4	82.1	92.1	97.0	97.1	90.0	90.1	89.1
TunLayer-1	94.1	91.7	93.4	94.9	62.0	64.9	77.0	91.6	95.3	92.5	90.0	91.2	87.3
TunLayer	94.1	91.7	93.4	94.9	62.0	64.9	77.0	91.7	95.3	92.6	90.0	91.2	87.3
TunFusLayer	96.6	93.0	95.4	96.0	68.5	67.0	80.8	92.9	98.3	96.7	90.6	91.1	89.5
Layer-3	96.3	91.7	95.4	95.3	65.0	63.8	75.7	89.2	99.0	97.7	88.3	90.4	87.9
DCT	99.1	95.0	98.6	**97.8**	83.5	73.4	90.9	97.5	**100**	98.9	90.7	91.6	93.1
g-DCT	96.9	91.0	95.6	94.0	66.0	70.2	83.2	92.5	99.0	94.8	89.7	90.1	88.6
PCA	98.1	94.8	98.4	97.3	81.5	75.3	91.5	97.5	99.7	99.4	92.3	**93.4**	93.3
LBPCHI	98.1	93.3	98.4	96.7	78.5	70.1	90.9	93.3	99.3	98.7	92.7	93.3	91.9
CHI	97.8	93.0	94.2	92.5	67.5	74.6	82.1	90.8	98.7	96.0	92.0	91.4	89.2
CoOC	98.1	94.6	97.2	97.5	80.5	71.1	85.9	94.2	98.7	98.8	92.3	92.4	91.8
GMTP	99.1	94.1	**99.0**	97.3	81.0	73.5	91.2	95.8	99.7	99.2	**93.7**	92.8	93.0
GEP	**99.4**	94.3	98.8	**97.8**	79.0	73.7	91.7	97.5	99.7	98.9	**93.7**	92.4	93.0
DCT+PCA	98.8	95.2	98.6	97.6	83.5	76.6	92.5	**98.8**	99.7	**99.6**	92.3	93.0	93.9
DCT+GMTP	98.8	**95.1**	**99.0**	97.6	85.0	74.6	92.5	98.3	**100**	**99.6**	92.7	92.8	93.8
(DCT+GMTP)-2	99.1	94.3	98.4	98.0	**87.5**	74.6	92.8	97.5	**100**	99.4	92.3	92.6	93.9
DCT+PCA+GMTP	98.8	94.5	**99.0**	97.6	84.0	76.7	**93.1**	98.3	99.7	**99.6**	92.3	93.1	93.9
(DCT+PCA+GMTP)-2	98.8	94.2	98.6	97.8	86.5	76.3	92.3	98.3	100	99.4	92.3	93.2	**94.0**
SFFS(20)	98.8	94.5	98.8	97.5	85.0	**76.8**	**93.1**	98.3	100	99.6	92.3	92.2	93.9
SFFS(10)	98.5	94.6	98.6	97.3	84.5	73.9	**93.1**	99.2	100	99.4	91.7	91.1	93.5

**Table 2:** Performance using GoogleNet (Bold face indicates best performances).

Method	GoogleNet												Avg
	CH	HE	LO	TR	RN	TB	LY	MA	LG	LA	BG	LAR	
CNN	96.3	88.4	94.4	92.7	40.5	61.2	72.0	86.7	94.3	89.5	89.3	88.3	82.8
Ens15CNN	97.8	93.7	97.6	96.0	55.5	68.9	74.4	88.3	95.0	84.7	94.6	92.8	86.6
TunLayer-3	97.8	91.4	97.2	94.4	63.0	64.3	79.7	87.1	97.7	95.4	91.0	90.7	87.5
TunLayer-2	97.5	90.9	96.0	93.3	64.5	64.2	77.9	84.6	97.0	95.8	92.0	91.0	87.1
TunLayer-1	96.0	89.3	95.0	93.3	43.0	64.0	74.4	86.2	97.3	92.9	90.7	88.8	84.2
TunLayer	96.0	89.3	95.0	93.3	43.0	64.0	74.4	86.2	97.3	92.9	90.7	88.9	84.2
TunFusLayer	96.9	90.3	95.6	94.5	55.0	64.7	77.9	87.1	97.7	96.2	92.3	90.0	86.5
DCT	97.5	92.3	97.2	94.0	64.0	71.2	79.7	90.8	99.7	97.7	91.7	91.7	89.0
g-DCT	95.1	85.4	89.6	84.9	53.5	67.6	71.5	80.0	97.7	92.0	82.0	89.5	82.4
PCA	97.2	91.9	97.0	94.5	55.5	71.5	77.6	89.2	99.0	96.9	92.7	91.8	87.9
GMTP	98.1	92.4	97.6	95.3	67.5	70.7	80.8	89.2	99.0	**98.1**	93.3	91.7	89.5
GEP	**98.5**	92.8	**98.0**	95.3	66.0	70.1	80.5	91.2	98.7	**98.1**	**93.7**	92.0	89.6
DCT+PCA	96.9	92.4	97.6	95.3	62.0	71.9	78.9	90.0	**99.7**	97.3	91.7	91.6	88.8
DCT+GMTP	97.5	92.6	**98.0**	95.3	66.5	71.4	81.3	89.2	**99.7**	97.7	93.0	92.0	89.5
(DCT+GMTP)-2	96.9	**93.1**	**98.0**	94.4	**68.5**	**72.6**	**83.2**	**92.1**	**99.7**	97.7	92.7	**92.4**	**90.1**
DCT+PCA+GMTP	96.9	92.3	**98.0**	**95.5**	64.5	71.8	79.5	89.2	99.3	97.5	93.0	91.7	89.1
(DCT+PCA+GMTP)-2	97.2	93.0	97.0	94.2	63.5	72.2	81.6	90.4	**99.7**	97.3	92.0	92.0	89.2

**Table 3:** Performance in the Virus data set to test generalizability.

Method		DenseNet201	ResNet50	DenseNet201+ResNet50
CNN	---	81.60	77.13	82.53
TunLayer-3	LibSVM	86.73	81.47	86.00
TunLayer-2		84.07	81.00	84.67
TunLayer-1		81.67	79.80	83.33
TunLayer		81.67	79.80	83.33
TunLayer-3	FitEcoc	85.67	83.73	85.67
TunLayer-2		83.00	80.80	84.07
TunLayer-1		81.00	79.27	81.67
TunLayer		81.00	79.27	81.67
DCT-2		87.20	87.73	89.07
DCT-2	FitEcoc	86.73	86.73	88.13
(DCT+GMTP)-2	LibSVM	89.27	88.27	89.60
(DCT+GMTP)-2	FitEcoc	87.67	88.73	88.67
(DCT+PCA+GMTP)-2	LibSVM	88.93	---	---
(DCT+PCA+GMTP)-2	FitEcoc	87.67	---	---

**Table 4:** Comparison with the literature.

This Work	[41]	[42]	[43]	[44]	[40]	[45]	[46]
89.60	89.47	89.00	88.00	87.27	87.00 *	86.20	85.70

Note: the method notated with * combines descriptors based on both *object scale* and *fixed scale* images.

## Data Availability

All data sets are publicly available.

## Appendix A

The following table expands the acronyms used in this paper.

**Acronym**	**Expansion**
CHI	Chi-square feature selection
CNN	Convolutional neural networks
CoOC	Deep co-occurrence of deep representation
Conv	Convolutional layer
DCT	Discrete cosine transform
FC	Fully connected layer
GEP	Global entropy pooling
GMTP	Global mean thresholding pooling
LBPCHI	LBP combined with chi-square feature selection is labeled LB
LBP	Local binary patterns
PCA	Principal component analysis
SIFT	Scale invariant feature transform
SFFS	Sequential forward floating selection
SVM	Support vector machine
TEM	Transmission electron microscopy
